# Supplementary material for: Exploring Physical Activity Levels, Barriers, and Education Sources in People with Cancer Undergoing Chemotherapy
Source: Cancers (Basel). 2025 Sep 12;17(18):2987. doi: 10.3390/cancers17182987 (PMC12468570; doi:10.3390/cancers17182987)
Supplement: Supplementary file 1 [file cancers-17-02987-s001.zip › cancers-3846252-supplementary.docx]

Appendix A – Supplementary Table

**Table S1:** CVI scores for questionnaire development

|  | **OC1** | **OC2** | **OC3** | **OC4** | **OC5** | **OC6** | **OC7** | **OC8** | **I -CVI** |
| --- | --- | --- | --- | --- | --- | --- | --- | --- | --- |
| 1. How many times in the past 7 days have you performed resistance exercise (strength training)? Where did you do this? | 4 | 4 | 4 | 4 | 4 | 4 | 4 | 4 | 1 |
| 2. I feel I have been provided with sufficient education regarding physical activity during my treatment. | 4 | 4 | 4 | 4 | 4 | 4 | 4 | 4 | 1 |
| 3. I would like more information about physical activity during my treatment. | 4 | 4 | 4 | 4 | 4 | 3 | 4 | 4 | 1 |
| 4. I have been referred to an Exercise specialist during my treatment. | 4 | 4 | 4 | 4 | 4 | 3 | 4 | 4 | 1 |
| 5. I have noticed a decrease in my strength, energy levels and ability to do tasks around the house. | 3 | 4 | 4 | 4 | 2 | 4 | 4 | 4 | 0.9 |
| 6. I have received education regarding physical activity from: (circle all that apply) | 3 | 3 | 4 | 4 | 4 | 3 | 4 | 4 | 1 |
| 7. Things that limit me exercising are: (circle all that apply) | 4 | 4 | 4 | 4 | 4 | 4 | 4 | 4 | 1 |
|  |  |  |  |  |  |  |  |  |  |
|  |  |  |  |  |  |  |  | **S-CVI** | 0.9 |
| ^OC = Oncology cliniciam^  ^I-CVI – Individual Content Validity Index^  ^S-CVI – Scale Content Validity Index^ |  |  |  |  |  |  |  |  |  |

**Disclaimer/Publisher’s Note:** The statements, opinions and data contained in all publications are solely those of the individual author(s) and contributor(s) and not of MDPI and/or the editor(s). MDPI and/or the editor(s) disclaim responsibility for any injury to people or property resulting from any ideas, methods, instructions or products referred to in the content.
